# Reproducibility of echocardiographic measurements of left ventricular systolic function: a systematic review and meta-analysis comparing artificial intelligence and clinician estimates

**DOI:** 10.1093/ehjdh/ztaf145

**Published:** 2025-12-11

**Authors:** Rebecca Roberts, Leigh Sanyaolu, Christina Sam, Daniel Farewell, Adrian Edwards, Rhodri H Davies

**Affiliations:** Division of Population Medicine, Cardiff University, 3rd floor, Neuadd Meirionnydd, Heath Park, Cardiff CF14 4XN, Wales, UK; Division of Population Medicine, Cardiff University, 3rd floor, Neuadd Meirionnydd, Heath Park, Cardiff CF14 4XN, Wales, UK; Division of Population Medicine, Cardiff University, 3rd floor, Neuadd Meirionnydd, Heath Park, Cardiff CF14 4XN, Wales, UK; Division of Population Medicine, Cardiff University, 3rd floor, Neuadd Meirionnydd, Heath Park, Cardiff CF14 4XN, Wales, UK; Division of Population Medicine, Cardiff University, 3rd floor, Neuadd Meirionnydd, Heath Park, Cardiff CF14 4XN, Wales, UK; Institute of Cardiovascular Medicine, University College London, 74 Huntley Street, London WC12 6BT, England, UK; Cardiology Department, University Hospital of Wales, Heath Park, Cardiff CF14 4XW, Wales, UK

**Keywords:** Artificial intelligence, Echocardiography, Reproducibility, Ejection fraction, Global longitudinal strain, Meta-analysis

## Abstract

Echocardiography underpins the diagnosis and management of cardiovascular disease, yet measurement variability can influence treatment decisions. Artificial intelligence (AI) may standardize interpretation, but its reproducibility and clinical impact require systematic evaluation. To compare the reproducibility of AI-derived and clinician-derived measurements of left ventricular (LV) systolic function, specifically global longitudinal strain (GLS) and ejection fraction (EF), in adults. We searched Medline, Embase, Web of Science, and CENTRAL from inception to May 2025 for peer-reviewed studies assessing the reproducibility of AI-derived EF and/or GLS from two-dimensional (2D) or three-dimensional (3D) transthoracic echocardiography. Reporting quality was assessed with the Checklist for Artificial Intelligence in Medical Imaging (CLAIM). Random-effects meta-analyses of intraclass correlation coefficients (ICCs) and Bland–Altman plots compared reproducibility of AI- and clinician-derived measures Nineteen studies (17 984 participants; mean age 59 ± 8 years, 52.8% male) were included. Mean CLAIM adherence was 72.9%. Pooled ICCs demonstrated high reproducibility for both AI- and clinician-derived EF and GLS. Bland–Altman analyses showed limits of agreement of −13.4% to +12.7% for 2D EF and −4.3% to +2.3% for 2D GLS. 3D EF was slightly better, showing pooled limits of agreement of 11.26–12.61%. The pooled mean absolute differences (MAD) were 5.17% for 2D EF, 5.27% for 3D EF, and 1.32% for 2D GLS. AI-derived GLS and 3D EF achieve reproducibility comparable to, or exceeding, clinicians’ estimates. However, the limits of agreement between clinician and AI estimates are sufficiently wide that reclassification is possible around key thresholds, which could affect patient management decisions. Large-scale, real-world validation remains essential to confirm generalizability.

## Graphical Abstract

**Graphical Abstract:**
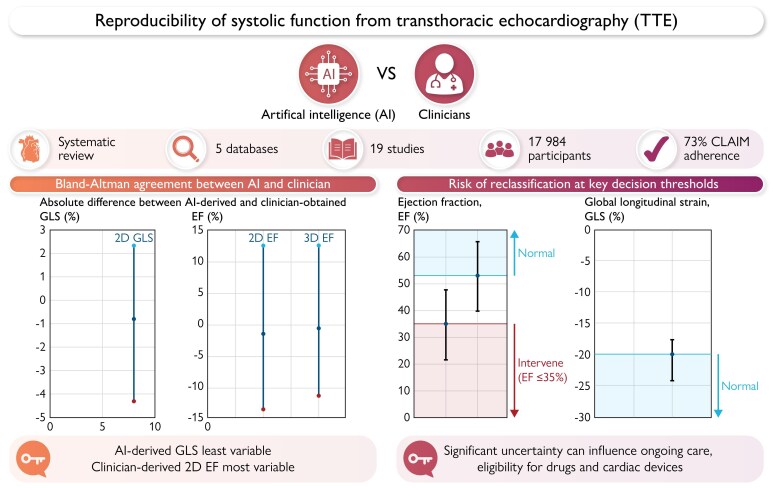

## Introduction

Cardiovascular disease (CVD) remains the leading cause of mortality worldwide, responsible for 18 million deaths per year.^1^ By 2050, the global burden of CVD is expected to increase substantially. It is estimated that the prevalence of CVD will increase by 90% and result in a 55% increase in disability-adjusted life years and a 73% increase in mortality.^2^ The projections place increasing demand on diagnostic imaging services for CVD, including echocardiography.

Whilst it is a cornerstone of clinical cardiology and integral to decision-making, echocardiography has limitations. Manual or semi-automatic image analysis is prone to intra-observer and inter-observer variability and is time-consuming.^3–6^ Additionally, it requires a highly skilled workforce. Globally, pressure on echocardiography services is rising, with national-level data from the UK revealing that an estimated 1.7 million echocardiography scans are requested annually, exceeding current service capacity.^7^ There is, therefore, an increasing interest in the application of artificial intelligence (AI) in this field. AI has shown promise in echocardiography, including enhanced diagnostic accuracy, operational efficiency, cost-effectiveness, and improved reproducibility.^8^ By reducing the variability introduced by differences in operator expertise, patient anatomy, and equipment, AI may help improve the consistency of echocardiographic measurements.^9^

However, whilst AI may offer a more consistent and reproducible alternative to manual measurements, its integration into clinical practice remains challenging. Barriers include clinician resistance, ethical concerns, and its compatibility with existing clinical workflow.^8,9^ Thus, the aim of this systematic review is to determine how reproducible echocardiographic measurements of left ventricular function obtained using AI are compared with those calculated by clinicians.

## Methods

### Study design

This was a systematic review and meta-analysis assessing the reproducibility of echocardiographic measurements of left ventricular function obtained using AI compared with those made by clinicians. All aspects of the review were conducted and reported in accordance with the Preferred Reporting Items for Systematic Reviews and Meta-Analyses (PRISMA) 2020 reporting guideline.^10^ The protocol for this systematic review is published on PROSPERO (ID: CRD42023477388).^11^ As this study involves secondary analysis of published data, ethical approval was not required. All data used were de-identified and included studies had ethical approval from their respective institutions.

### Study selection

We searched Medline via Ovid using Medical Subject Headings (MeSH terms) combined with Boolean operators, ‘AND’, ‘OR’. To reflect the full evolution of AI in echocardiography, we did not apply publication date restrictions and searched databases from inception.

We co-developed our search with an information scientist using search terms related to artificial intelligence, echocardiography, and reproducibility, also utilizing the search strategies of existing Cochrane reviews and an AI search filter for Medline.^12,13^

Our search (Appendix A) was developed in Medline and retrieved records from inception to 14 May 2025. This search was adapted to three additional electronic databases, Embase via Ovid, Web of Science, and CENTRAL, to capture relevant literature.

Our pilot search identified a substantial number of relevant studies. Only peer-reviewed publications were included. Preprints were excluded to ensure that all included studies had undergone peer review, maintaining methodological and reporting quality, and minimizing the risk of including unvalidated findings that could undermine the reliability of our conclusions.

After removing duplicate records using EndNote (Clarivate) and Rayyan (Qatar Computing Research Institute, QCRI), all remaining records underwent title and abstract screening. RR conducted the initial screening based on the predefined eligibility criteria (*Table 1*). A second reviewer (CS) independently screened a random 10% sample to ensure fairness in inclusion. The inter-rater reliability, measured using Cohen’s kappa (*κ* = 0.83), indicated ‘almost perfect’ agreement, so further dual screening was not performed.^14^

**Table 1:** Eligibility criteria

Inclusion criteria	Exclusion criteria
Papers available in English.	Grey literature.
Peer-reviewed studies. Primary research.	Reviews, systematic reviews, meta-analyses, editorials, letters, conference posters, case reports, opinions, book chapters, letters, case series, commentaries, conference papers or posters, proceedings, dissertations, and thesis submissions.
Reporting the reproducibility of echocardiographic measurements of left ventricular function using AI.	Not reporting the reproducibility of echocardiographic measurements of left ventricular function using AI.
2D- or 3D-transthoracic echocardiography.	Studies using other types of imaging techniques.
Comparator group is clinicians or specialist physiologists.	No comparator group, or comparator group not involving clinicians or specialist physiologists.
Adult human participants (18 and over) without congenital heart disease.	Participants under 18 or those with congenital heart disease.
Reporting of at least one reproducibility metric, including the coefficient of variation (CoV), Cohen's *κ*, Bland–Altman limits of agreement (LoA), and intraclass correlation coefficient (ICC).	No reporting of reproducibility statistics.

**Figure 1:**
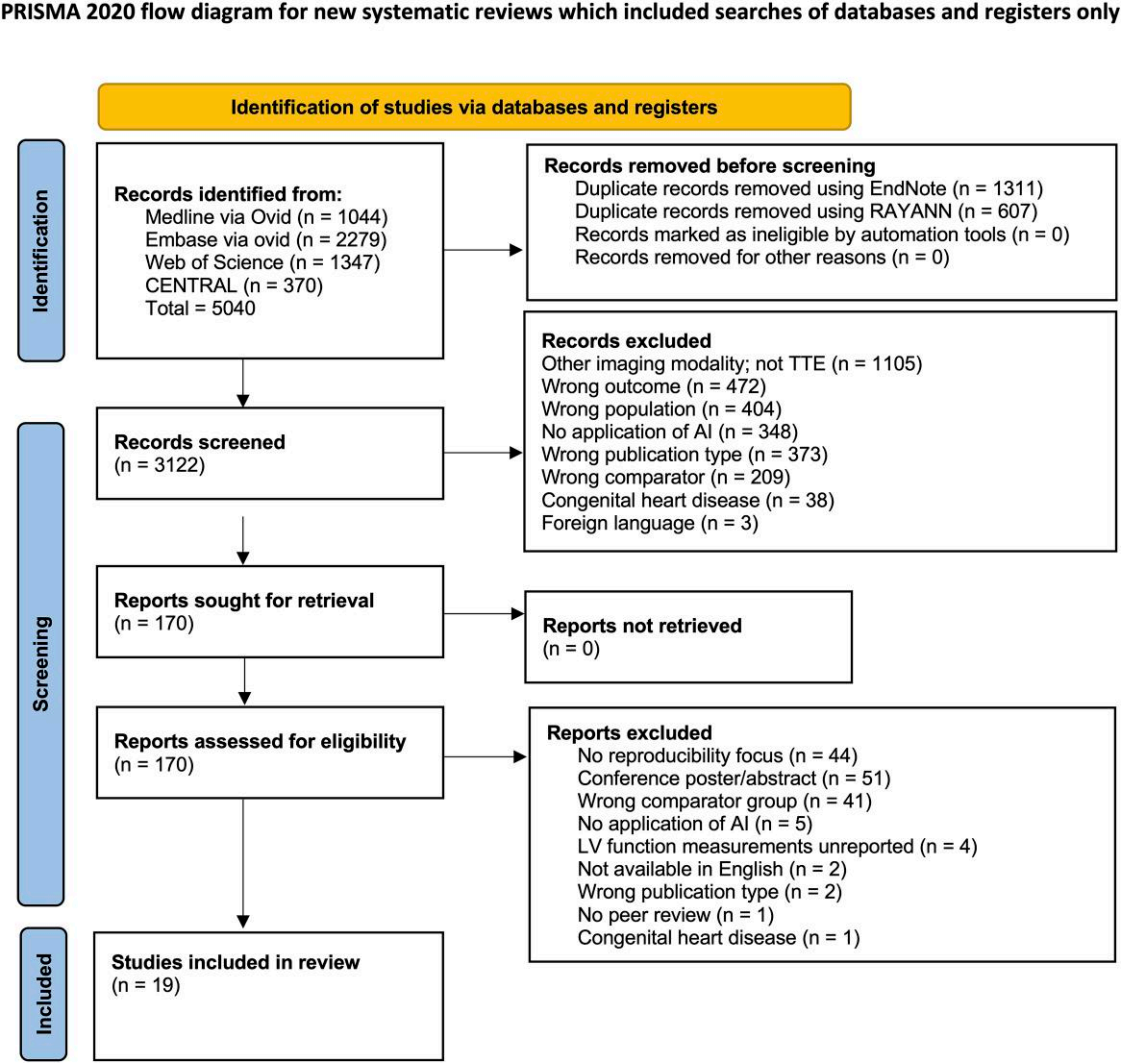
PRISMA flow diagram depicting study selection process, including records from searches in December 2024 and May 2025.^10^

Disparities between reviewers (C.S. and R.R.) were discussed and a consensus was reached for all conflicts and third-party arbitration was not required. At both stages, all excluded studies and reasons for exclusion were documented on the PRISMA 2020 flow diagram (*Figure 1*). Full-text screening was performed by R.R. for potentially eligible studies, in consultation with R.D., an imaging cardiologist with expertise in AI, to ensure clinical eligibility criteria were met.

### Data extraction

A summary of the extracted information is presented in *Table 2*. Subsequently, data were assessed for suitability for meta-analysis, depending on the level of heterogeneity across studies, principally the type of reproducibility metrics presented.

**Table 2:** Data extraction

Study characteristics	Title, author, publication year, journal, nationality.
Methodology	Study design, study population echocardiographic modality, comparator methods.
AI usage	Type of AI model (DL, ML), validation.
Reproducibility metrics	ICC, Bland-Altman Limits of Agreement, CoV, Cohen's κ.
Strengths and limitations	As acknowledged by the study.
Conclusion	Overall conclusion, study funding, other strengths and limitations, conflict of interest.

DL = deep learning; ML = machine learning; CoV = coefficient of variation; ICC = intraclass correlation coefficient.

### Quality assessment

We used the 42-item Checklist for Artificial Intelligence in Medical Imaging (CLAIM) to assess the quality of evidence.^15^ Whilst CLAIM is primarily a reporting tool rather than an appraisal tool, it offers a structured framework for evaluating the transparency and methodological rigour of AI-based medical imaging studies in the absence of a widely accepted AI risk of bias tool specific to reproducibility studies.

Quality assessment evaluated each study against the key CLAIM domains:

- Study design and patient selection.
- AI model development and validation.
- Comparator and ground truth.
- Performance metrics and reproducibility.
- Clinical relevance and generalizability.

The mean and standard deviation (SD) of CLAIM scores were also calculated to summarize the overall reporting quality.

### Data synthesis

Reproducibility refers to the consistency of repeated measurements on the same subject under varying conditions and is essential for clinical reliability.^9^ It can be assessed using metrics such as the coefficient of variance (CoV), Cohen's *κ*, Bland–Altman limits of agreement (LoA), and the Intraclass Correlation Coefficient (ICC).^9^

The ICCs and Bland–Altman analyses were consistently reported across studies, enabling meta-analysis. Due to methodological and clinical differences across studies, including variations in AI models, study populations, and comparator groups, a random-effects model was used to allow for variation in the underlying effect sizes.^16^

To conduct the ICC meta-analysis, we first estimated the variance using the formula by Bhat and Beretvas^17^:


$$ICC\;Variance=(2\;\times \;(1-ICC{)}^{2}/(n-1))$$

where *n* = sample size

Anticipating significant heterogeneity between studies, we conducted separate random-effects meta-analyses using the standard error to obtain pooled inter-observer, intra-observer, inter-technique, and AI-derived ICCs.^18^ Inter-observer ICCs reflect agreement between different human observers; intra-observer ICCs measure the consistency of the same observer over time; inter-technique ICCs assess reproducibility between clinician and automated measurements, and AI-derived ICCs reflect the consistency of repeated measurements made by the AI model. The results are summarized in tables within the results section.

Despite the popularity of Bland–Altman plots, meta-analytical methods for synthesizing their results are underdeveloped.^19^ Hence, we followed the framework proposed by Tipton and Shuster to conduct an inverse-variance weighted meta-analysis of the bias.^18,19^ This process involved calculating the standard deviation of differences, assessing normality, determining inverse-variance weights, and computing both the weighted bias and the mean weighted bias, along with their standard error and 95% confidence intervals (CI).^19^ The full details and equations used are available in Appendix B.

The standard error for the LoA was estimated as^20^:


$$SE=\mathrm{SD}\;x\sqrt{\frac{1}{n}+\frac{{\left(1.96\right)}^{2}}{2(n-1)}}\;\;\;\;\;\;\;\;\;$$

where SD = standard deviation of differences; *n* = sample size

Two separate random-effect meta-analyses for the upper and lower LoA were performed to obtain a pooled estimate. The results are displayed graphically, illustrating the individual study estimates alongside the meta-analysed result, with 95% CI and standard errors for each data point.

All meta-analyses were conducted using the statistical package SPSS (version 29.0.2.0, IBM). Bland–Altman analyses are presented using Microsoft Excel (version 16.87, Microsoft Corp).

In addition to the meta-analyses, we generated graphs to contextualize measurement variability in relation to guideline-defined clinical thresholds for EF and GLS. For each study, Bland–Altman limits of agreement were extracted and entered into Excel to illustrate agreement between clinician and AI estimates at decision-relevant cut-offs. The mean absolute difference (MAD) was calculated from the standard deviation of differences, approximating the average magnitude of error under the assumption of normally distributed differences, using the formula^21^:


MAD=(2xSD)÷π

We further calculated mean absolute percentage difference (MAPD) by dividing the MAD by the relevant threshold and multiplying by one hundred, thereby expressing error size relative to clinical thresholds.

## Results

### Study selection

The search identified 5,040 records (Medline via Ovid: 1,044, Embase via Ovid: 2,279, Web of Science: 1,347, CENTRAL: 370). After removal of duplicates (*n* = 1918), 3,122 records remained for initial title and abstract screening.

A total of 2,952 records did not meet the eligibility criteria, leaving 170 studies for full-text screening. Of these, a further 151 were excluded and 19 studies were included in our systematic review. A PRISMA 2020 flow diagram illustrating the study selection process is shown in *Figure 1*.

### Study characteristics

Of the 19 studies, 10 assessed the reproducibility of AI-derived EF, 4 assessed AI-derived GLS, and 5 evaluated both. Participant demographics were available for 18 of these studies and enrolled 17,984 participants^22–37^. Of these, 9487 (53%) were male. The mean age was 59 ± 8 years. *Table 3* provides a summary of study characteristics, including sample size, AI model type, the clinical comparator (e.g. expert clinician, sonographer) and CLAIM score.

**Table 3:** Summary of study participant demographics and CLAIM scores across all included studies

Measure of LV function	2D/3D TTE	Study	Sample	Study characteristics					AI model	Comparator	CLAIM score
				Male	Female	Age	Arrhythmia	VHD			
LVEF	2D	Asch *et al*. (2019)^37^	99	62	37	66.0	n.d	n.d	ML algorithm (AutoEF, Bay Labs Inc.)	Three echocardiographers	28
	2D	Kim *et al*. (2022)^34^	500	251	249	36.2	n.d	0	Three DL algorithms (U-net, Res-U-net, Dense-U-net)	Two sonographers	26
	3D	Medvedofsky *et al*. (2018),^31^	180	119	61	57.0	29	20	ML, HeartModel (Philips Healthcare)	*‘Trained personnel at a highly experienced Core Laboratory (CL)’*	35
	2D	Mor-Avi *et al*. (2023),^30^	12	n.d	n.d	n.d	n.d	n.d	DL algorithm	Ten echocardiographers	30
	2D	Morbach *et al*. (2024),^29^	4965	2404	2561	54.9	n.d	n.d	ML within a federated learning framework	*‘Trained and internally certified personnel performed measurements’*	37
	2D/3D	Myhr *et al*. (2018),^28^	100	38	62	67.0	10	37	AutoEF (2D) 4D Auto LVQ (3D)	One sonographer	31
	2D	Olaisen *et al*. (2024),^25^	3282	1865	1417	59.5	322	60	DL based on U-net architecture for segmenting LV endocardium, myocardium & atrium	Dataset 1: three cardiologists Dataset 2: four ‘experienced operators’ Dataset 3: two cardiologists Dataset 4: sonographer, cardiologist	29
	2D	Sveric *et al*. (2023),^38^	889	542	347	71.0	223	181	DL algorithm, LVivo Seamless™	Cardiologist	30
	2D	Li *et al*. (2025),^39^	2461	1329	1132	52.4	n.d	n.d	DL custom modular model based on YOLOX	*‘Two highly experienced doctors, each possessing over 10 years of clinical expertise in cardiac ultrasound…’*	33
	2D	Lin *et al*. (2024),^40^	2613	1163	1450	56	n.d	n.d	QHAutoEF, integrating DL and transformers	Multiple senior echocardiographers.	40
GLS	2D	Nyberg *et al*. (2024),^26^	80	48	32	61.0	n.d	n.d	DL-method based on point tracking.	*‘Three experienced observers’*	26
	2D	Rogstadkjernet *et al*. (2024),^23^	605	372	233	63.4	n.d	143	DL segmentation model, EfficientNetB1	Echocardiographers	34
	2D	Salte *et al*. (2023),^22^	72	42	30	63.5	n.d	n.d	DL-method	Four echocardiographers	28
	2D	Kuwahara *et al*. (2024),^32^	94	25	69	69.0	n.d	n.d	U-Net DL for endocardial segmentation, *Caas Qardia 1.1 software*	*‘Experienced examiners’*	34
LVEF, GLS	2D	Jang *et al*. (2024),^36^	632	531	101	59.3	n.d	n.d	3D DL segmentation & motion estimation	Sonographers, echocardiographers	26
	2D	Knackstedt *et al*. (2015),^33^	255	153	102	50.3	Excluded.	4	ML-algorithm, AutoLV	*‘Expert investigator (level 3 training in echocardiography; C.K., A.F., L.B., P.S.)’*	33
	2D	Lafitte *et al*. (2025),^41^	894	510	384	64.8	n.d	n.d	Deep learning, Us2.ai	*‘Operators with three experience levels (nurses, residents and experts)’*	27
	2D	Jiang *et al*. (2023),^35^	142	2	140	59.0	Excluded.	n.d	DL, Ligence Heart (version 2)	*‘BSE-accredited or similarly experienced operator manually adjusted the ROI”*	29
	2D/3D	Myhre *et al*. (2024),^27^	109	32	77	56.0	Excluded.	n.d	2D: Us2.ai 3D: Heartmodel3D, echo analysis	*‘Experienced operator had EACVI transthoracic echocardiography certification or an echocardiography experience of more than 10 years”*	26

AI = artificial intelligence; EF = ejection fraction, GLS = global longitudinal strain; LV = left ventricle; TTE = transthoracic echocardiography; VHD = valvular heart disease; DL = deep learning; machine learning = machine learning; CLAIM = checklist for artificial intelligence in medical imaging; n.d = not disclosed.

Heterogeneity of findings within each random effects model ranged from 45 to 100% and thus was generally substantial (Appendix C).

### Study quality

The mean CLAIM score across all studies was 30 ± 4 out of a maximum score of 42 (72.9%), indicating moderate to high adherence with reporting standards. The highest mean percentage completion is seen in the title/abstract (100%), introduction (100%), study design (100%), and discussion (100%) domains. The poorest are seen in other information (35.1%) and training (45.6%) domains. A summary of CLAIM domain scores across all included studies can be found in Appendix D.

### Ejection fraction (EF)

To contextualize the pooled reproducibility estimates, Supplementary material online, *Table S1* presents the raw mean EF values and their standard deviations as reported by clinicians and AI across the studies included in our review. AI-derived EF values were broadly comparable to those of clinicians, with a slight tendency towards lower estimates with similar variability.

ICCs for ejection fraction (EF) were reported in 10 studies using 2D TTE, with no data available for 3D TTE. Only one study reported an AI-derived ICC of 0.92 (95% CI: 0.900, 0.936), precluding meta-analysis.^37^ We undertook meta-analyses of human inter-observer, human intra-observer, and inter-technique ICCs for comparison (*Table 4*). Although AI-specific data were limited, the reported AI ICC (0.92) exceeded those of others, suggesting that AI may yield more reliable EF measurements from 2D TTE images than manual methods performed by either the same or different clinicians.

**Table 4:** Pooled intraclass correlation coefficients (ICCs) with 95% CI for manually derived EF and inter-technique agreement from 2D TTE images

	Pooled ICC (95% CI)
Manually derived EF inter-observer agreement^27,33,34^	0.83 (0.76, 0.91)
Manually derived EF intra-observer agreement^27,28,33,34,37^	0.88 (0.82, 0.95)
Inter-technique agreement^28,33,36–41^	0.85 (0.80, 0.88)

**Figure 2:**
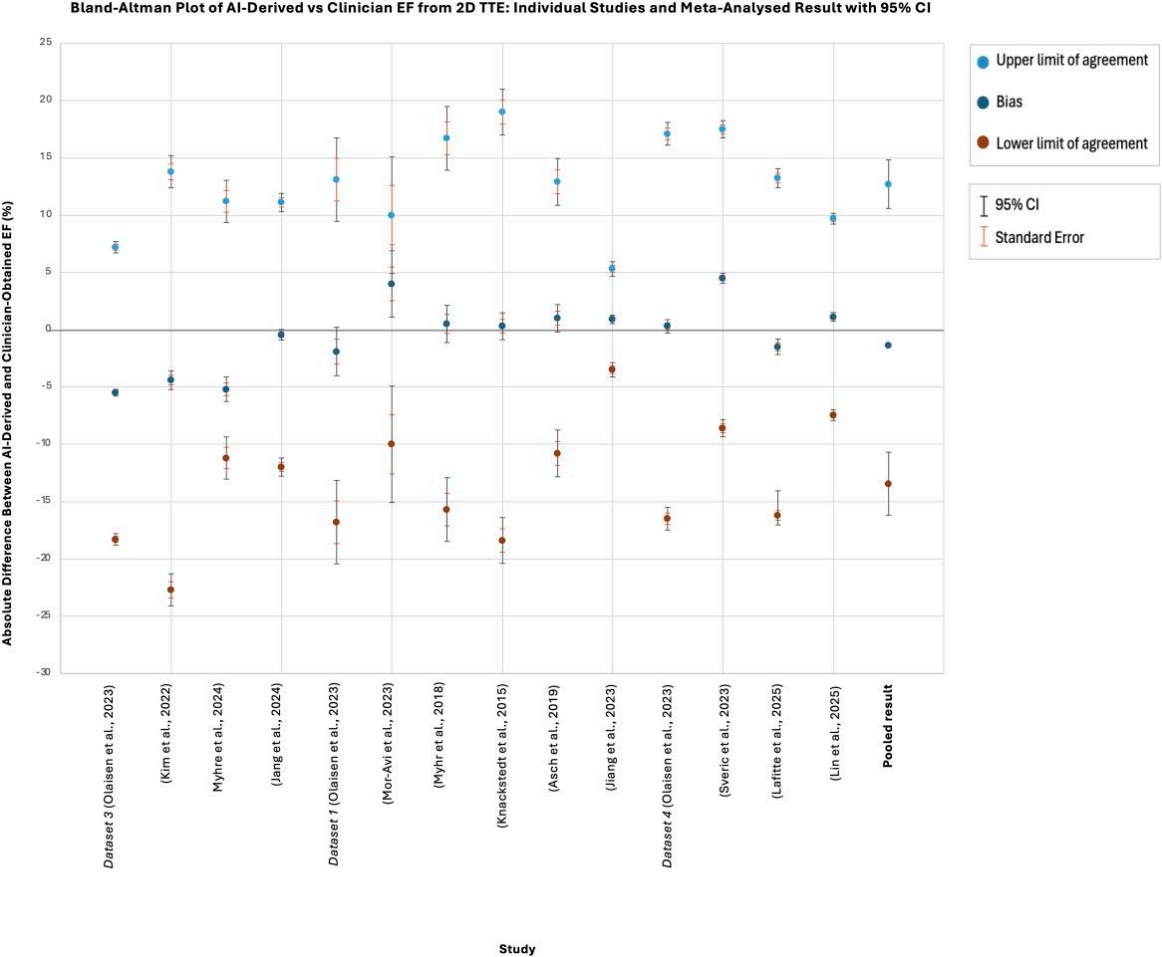
Bland–Altman plot presenting agreement between AI-derived and clinicians' EF from 2D TTE images. Individual study results and pooled results from meta-analysis shown.

*Figure 2* presents the Bland–Altman results depicting agreement between AI and clinicians’ EF values from 2D TTE images. Across the included studies, bias values ranged from −5.5% to 4.5% (total range 10.0%). The pooled mean bias was −1.4% (95% CI: −1.35, −1.42), consistent with the raw data in Supplementary material online, *Table S1,* which shows that AI generally produces lower EF estimates than clinicians. The pooled LoA are wide, ranging from −13.44% (95% CI: −16.19, −10.69) to +12.7% (95% CI: 10.59, 14.82), indicating significant variability in AI-derived EF measurements relative to the clinicians’ reference point.

**Figure 3:**
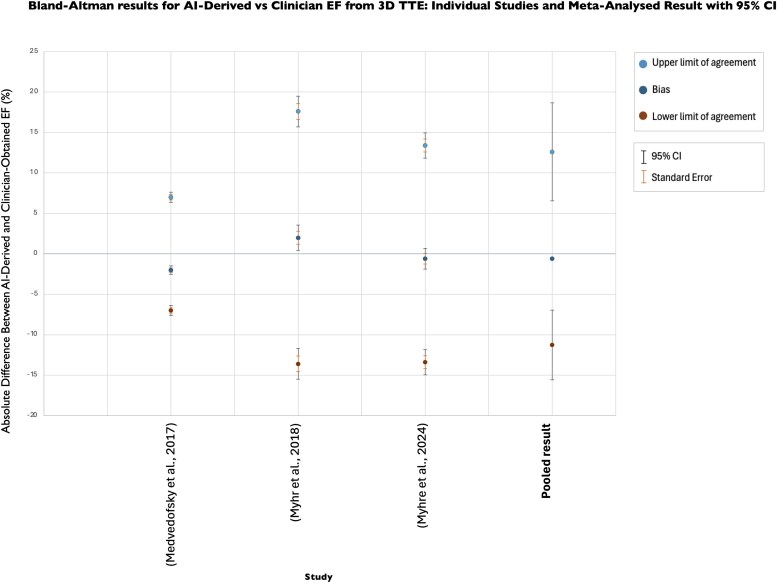
Bland–Altman plot presenting agreement between AI-derived and clinicians' EF from 3D TTE images. Individual study results and pooled results from meta-analysis shown.

*Figure 3* displays the Bland–Altman results showing the agreement between AI- and clinician-derived EF from 3D TTE images. Similarly, a pooled bias of −0.58% (95% CI: −0.72, −0.44) indicates that AI values are marginally lower than clinician-derived EF. Notably, the magnitude of the bias in 3D TTE is smaller than that observed with 2D TTE. Variability is also slightly reduced as reflected by marginally narrower pooled LoA for 3D EF, ranging from −11.26% (95% CI: −15.58, −6.95) to 12.61% (95% CI: 6.55, 18.67), indicating a slight improvement in consistency in AI-derived EF from 3D TTE compared with 2D TTE images.

**Figure 4:**
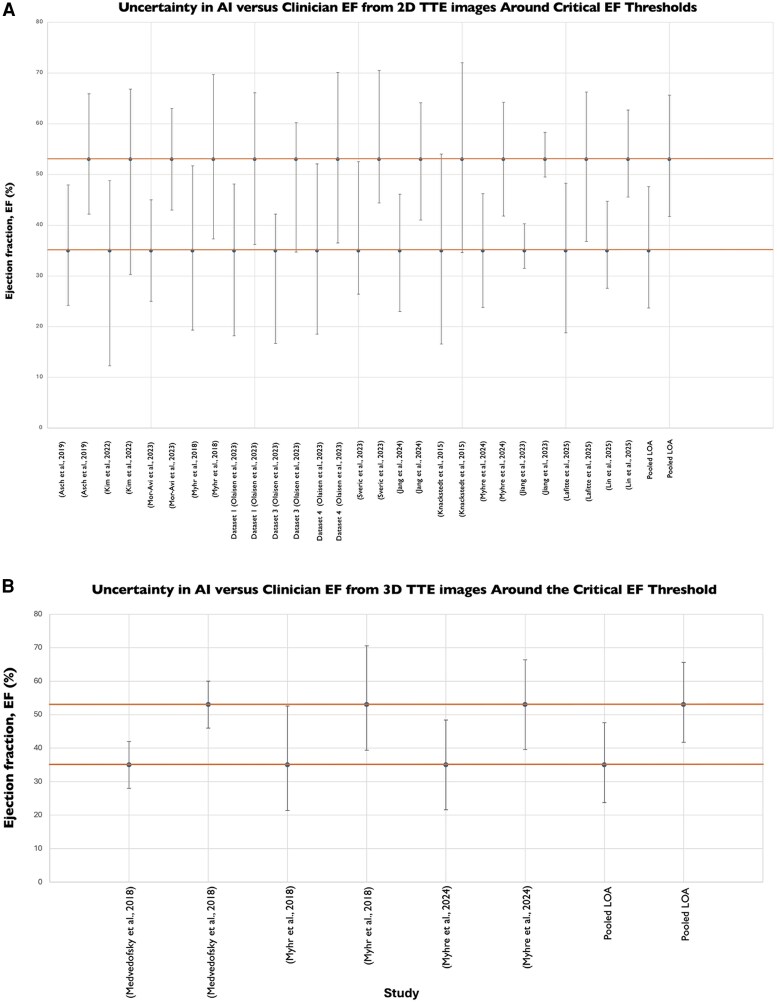
Uncertainty in AI-vs. clinician-derived ejection fraction (EF) measurements around guideline-defined clinical thresholds. (*A*) 2D TTE studies, (*B*) 3D TTE studies.

To contextualize these findings against clinical thresholds, we evaluated agreement between clinician- and AI-derived EF from both 2D and 3D TTE images at guideline-defined cut-off points (*Figure 4*). Normal left ventricular systolic function is defined as>52% in men and >54% in women, while an EF **<** 35% represents the threshold for considering cardiac resynchronization therapy (CRT) or primary prevention ICD implantation.^42,43^

For 2D TTE, *Figure 4A* demonstrates that variability between AI- and clinician-derived EF is considerable, with a MAD of 5.17%. This corresponds to MAPD of 14.8% at the 35% treatment threshold and 9.9% at the 53% ‘normal function’ cut-off. Discrepancies of this magnitude have the potential to alter clinical classification at clinical decision-making thresholds.

For 3D TTE, *Figure 4B* also shows considerable variability, with a MAD of 5.27%, corresponding to a MAPD of 15.1% at the 35% threshold and 9.9% at the 53% threshold. Although the magnitude of error is almost identical to that observed with 2D TTE, the persistence of variability across both modalities suggests that disagreement between AI and clinician assessment is unlikely to be a limitation of one imaging approach but rather reflects a systematic challenge in reproducibility.

### Global longitudinal strain (GLS)

Supplementary material online, *Table S2* summarizes the raw mean GLS values with standard deviations reported in the included studies from 2D TTE images. While broadly aligned with clinician measurements, AI-derived GLS tended to be marginally lower and demonstrated reduced variability.

*Table 5* presents the pooled ICC values with 95% CIs for agreement in GLS measurements between AI and clinicians’ values from 2D TTE images. Manual intra-observer had an ICC of 0.85 (95% CI: 0.77, 0.93) compared with ICC of 0.81 for AI (95% CI: 0.75, 0.87), both indicating good reproducibility. The overlapping but narrower confidence intervals suggest that AI’s consistency may be comparable to or potentially exceed that, manual intra-observer interpretation. Manual inter-observer agreement was lower with only moderate reproducibility (ICC = 0.75; 95% CI: 0.68–0.83), highlighting the greatest variability between clinicians’ assessments. The inter-technique ICC (ICC = 0.77; 95% CI: 0.65, 0.88) indicates moderate agreement; however, the wide confidence interval suggests uncertainty in this value.

**Table 5:** Pooled ICC (95% CI) for agreement in GLS measurements from 2D TTE, comparing AI-derived and manual methods

	Pooled ICC (95% CI)
AI-derived GLS agreement^22,32^	0.81 (0.75, 0.87)
Manually derived GLS inter-observer agreement^22,26,32,33^	0.75 (0.68, 0.83)
Manually derived GLS intra-observer agreement^22,26,33^	0.85 (0.77, 0.93)
Inter-technique agreement^26,27,35,36,41^	0.77 (0.65, 0.88)

**Figure 5:**
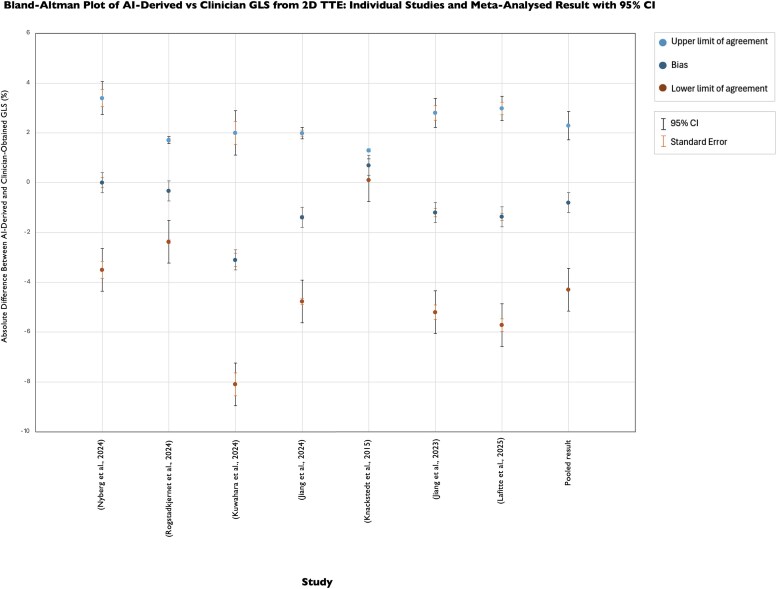
Bland–Altman plot presenting agreement between AI-derived and clinicians' GLS from 2D TTE images. Individual study results and pooled results from meta-analysis shown.

*Figure 5* presents the Bland–Altman results depicting agreement between AI and clinician-derived GLS from 2D TTE images. Seven studies reported Bland–Altman results and were included in the meta-analysis. The pooled mean bias was −0.80 (95% CI: −0.89, −0.76). Consistent with trends observed in AI-derived EF, AI produces lower estimates of GLS but nonetheless demonstrates excellent agreement with manual measurements. Furthermore, the pooled LoA are narrow and reflect low variability ranging from −4.30% (95% CI: −6.15, −2.28) to +2.30% (95% CI: 1.72, 2.86).

**Figure 6:**
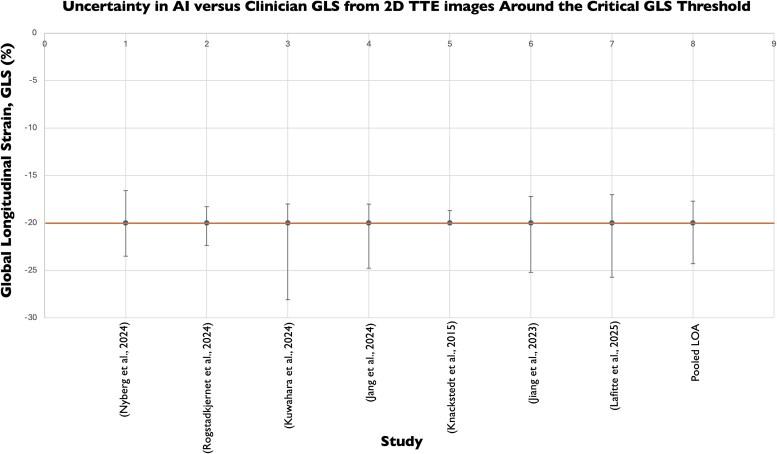
Agreement between clinician- and AI-derived global longitudinal strain (GLS) values from 2D transthoracic echocardiography (TTE) at guideline-defined clinical thresholds.

To contextualize these findings against clinical thresholds, we evaluated agreement between clinician- and AI-derived GLS from both 2D TTE images at the threshold for a normal GLS, 20% (*Figure 6*). We obtained a MAD of 1.32%, corresponding to a MAPD of 6.61%.

## Discussion

### Principal findings

This systematic review assessed the reproducibility of echocardiographic measurements obtained using AI, compared with those calculated by clinicians. The findings suggest that AI-derived GLS from 2D transthoracic echocardiogram (TTE) images and EF from 3D TTE images are reproducible. Measurement variability, evidenced by Bland–Altman LoA, is reported for AI-derived EF from 2D TTE, AI-derived EF from 3D TTE, and 2D AI-derived GLS, with each modality showing distinct limits of agreement.

AI-derived intraclass correlation coefficients (ICC) for both EF and GLS were high, and each exceeded human inter-observer ICC. While the manual intra-observer ICC for GLS was marginally higher than the AI-derived ICC, overlapping and narrower confidence intervals suggest that AI has the potential to at least replicate the level of consistency expected from a single trained operator. Since AI holds the potential to standardize measurements across clinicians, settings, and institutions, this level of reliability is clinically promising.

### Existing literature

These findings complement a growing body of literature reporting that AI can enhance reproducibility in echocardiography by reducing inter-observer variability inherent in manual measurements.^5,44^ Nonetheless, concerns remain that AI reproducibility is highly dependent on image quality, a limitation highlighted by several studies included in this review.^31,34,35,37,45^ Image quality can be affected by differences in equipment and patient-related factors, including body habitus, arrhythmia, and respiratory patterns, all of which may introduce noise and artefacts that reduce algorithmic accuracy and impact reproducibility.^46,47^

Similarly, the improved reproducibility observed in clinician-derived EF using 3D TTE compared with 2D TTE has been attributed to differences in image acquisition. 3D TTE offers more comprehensive volumetric data, reducing geometric assumptions and thereby improving consistency in image alignment and endocardial border detection.^48^ These same advantages likely contribute to greater reliability in AI-derived EF from 3D datasets as well.

Improvements in reproducibility with AI integration have also been observed in other cardiac imaging modalities, including cardiac MRI and CT, supporting the broader claim that AI can enhance standardization and reproducibility in cardiac imaging, provided that image quality is adequate.^49,50^

### Clinical implications

The considerable variation observed in clinicians’ 2D EF measurements has potential for profound clinical implications. EF is the gatekeeper for many drugs and devices, and any error in EF measurement has the potential to affect clinical decision-making.^51^  *Figures 4* and *6,* which illustrate agreement between clinician and AI EF estimates from 2D and 3D TTE images around clinical decision-making thresholds, highlight the risk that measurement variability may result in patient reclassification. For EF, even modest discrepancies could shift an individual across treatment boundaries, with potential consequences for device eligibility or for distinguishing normal from impaired systolic function.^42,43^

AI-derived GLS demonstrated high precision, with narrow LoA, outperforming clinicians’ inter-observer variability, which ranges from 5.4% to 8.6% in the literature.^52^ This higher degree of reproducibility is particularly encouraging given the growing reliance on GLS as a marker for early detection of subclinical myocardial dysfunction, particularly among patients receiving cardiotoxic chemotherapies such as anthracyclines or trastuzumab.^53,54^ Given that small, clinically meaningful changes in GLS can influence management, measurement precision is paramount. Greater variability between human observers may mask these subtle changes, misinforming clinical decision-making. In contrast, AI's ability to deliver more consistent measurements and reduce inter-observer variability may mitigate these challenges. Other key potential benefits include reducing reliance on a highly specialized workforce for image interpretation, thereby alleviating pressure on overstretched echocardiography services.^4^ Taken together, these findings suggest that AI-derived GLS may represent the most immediate avenue for clinical deployment, offering greater reproducibility than EF and reducing the risk of misclassification in scenarios where subtle changes matter.

Despite overall moderate to high adherence with the CLAIM reporting tool, the ‘training’ domain had the lowest average score. The training section covers details of the description of training data, labelling and ground truth, preprocessing, dataset splitting, handling of bias, and availability of the dataset.^15^ To ensure equity and safety, future policy must prioritize transparency in training data, which will also allow clinicians in other institutions to replicate and externally validate AI tools to assess reproducibility. An additional important implication is the potential for automation bias. There is evidence to suggest that clinicians often lack the technical expertise to critically assess AI, leading to unintentional misuse and overreliance on model outputs.^55,56^

### Current limitations of AI in echocardiography

Despite AI’s promise, several limitations must be acknowledged. It is well established that echocardiographic images differ substantially between ultrasound machines and manufacturers, as well as across image analysis software packages.^57,58^ While AI algorithms can standardize interpretation to reduce variability introduced during image analysis, they cannot yet overcome fundamental differences in raw image acquisition between vendors. With several global vendors routinely used in clinical practice, this variability poses a persistent barrier to universal application, as both AI- and clinician-derived measurements can be influenced by variability in acquisition and analysis hardware and software.^59,60^ Standardization of definitions, image acquisition protocols, analysis techniques, and reporting workflows, e.g. in speckle-tracking echocardiography, can help mitigate these discrepancies.^54^ Recent research has explored the use of adversarial AI models to minimize inter-domain variability, while training on federated datasets has shown promise in improving vendor-independence and cross-domain generalisability.^29,61,62^ The broader issue of generalizability across diverse populations and clinical settings is considered further in our Future Research section.

In addition, LVEF and GLS have no definitive ‘ground truth,’ so even highly reproducible AI estimates cannot be assumed to be fully accurate. Differences observed between AI and manual (or semi-automatic) measurements, therefore, reflect variability in the assessment methods rather than necessarily indicating under- or overestimation.

### Future research

Future studies should focus on the external validation of AI models across diverse populations, institutions, and vendor equipment. We included both true test–retest studies, where two separate imaging acquisitions were performed in the same patient, and studies that compared images acquired within a single session but from different cardiac cycles. While both approaches yield different images, test–retest studies are preferable, as they more closely reflect real-world clinical variability, including differences in probe placement, patient positioning, and operator technique. This is particularly relevant for the management of chronic cardiac disease, where serial echocardiographic assessment of LV function, often performed by different clinicians, is used to monitor long-term disease progression or treatment response in the same patient.^63^ In this context, evaluating test–retest reliability is essential to determine whether changes in cardiac function reflect true disease progression or are simply due to measurement variability.^63^

Furthermore, the consequence of AI training on predominantly male TTE images cannot be ignored and the impact of this needs to be explored. It is imperative to develop methods to mitigate the potential gender bias and promote equity in algorithmic training.

Yet, publishing the results of AI systems is only the first step towards clinical translation. The next stage requires demonstrating improvements in patient outcomes and establishing cost-effectiveness.^64^ Regulatory approval, prospective clinical validation, and integration into existing workflows remain substantial challenges; hence, most published AI algorithms are never implemented in practice.^65^

To support clinical integration, future research should focus on large-scale, prospective validation of AI models across diverse populations and settings, transparent reporting of training data, mitigation of bias, and demonstration of improvements in patient outcomes and cost-effectiveness. Collaboration between clinicians, industry, and regulators will be essential to ensure safe and effective implementation into routine echocardiographic practice.

### Strengths and limitations

To the best of our knowledge, this is the first systematic review to evaluate and meta-analyse the reproducibility of AI-derived echocardiographic measurements of left ventricular function. The methodology was rigorous, adhering to PRISMA 2020 guidelines, and our search strategy was co-developed with an information scientist. Included studies had moderate to high quality in reporting, with a mean CLAIM score of 31 ± 4 out of 42 (72.9%)**—**higher than the median score of 26 reported across 421 AI imaging studies from 1997–2024.^66^ A further strength lies in the novel application of meta-analytic methods for Bland–Altman analyses using Tipton and Shuster’s framework.^19^

Several limitations should also be acknowledged. In some studies, the level of experience of the clinical comparator was unclear, potentially introducing comparator bias as reproducibility is known to be influenced by the operator’s expertise.^41,67^ Evidence suggests that less experienced operators experience greater variability in measurement interpretation, consistency, and diagnostic accuracy.^67^

Furthermore, caution is warranted given the heterogeneity within the models (Appendix C), although this was to be expected given the relatively small number of included studies. As a result, estimates of heterogeneity are likely to be imprecise.^68^

Generalizability is a key limitation. Many AI algorithms were developed using single-centre, single-vendor datasets, which may limit their applicability across different clinical settings.^28,35,38^ Although the overall study population was slightly male-predominant (53%), individual studies varied significantly in sex distribution, with the highest discrepancy reflected by a 5:1 male-to-female ratio.^36^ This raises concerns regarding the applicability of findings to female patients, particularly in light of known sex-based differences in cardiac structure and function that may impact measurement reproducibility.^69^

Additionally, a significant number of studies were conducted retrospectively.^25,27,32,34–37^ Prospective studies including real-time validation are needed to evaluate how these algorithms perform when integrated into routine care.

We were unable to perform subgroup analyses by clinical condition, as very few conditions were specified in the original studies, and those that were reported were poorly defined. This is an important limitation given that differences in underlying pathology may substantially influence results and contribute to selection bias. This is particularly relevant for AI, which typically performs best when it has been trained on similar examples.^70^ For instance, if a model has not encountered an unusual morphology during training, such as a large apical aneurysm with associated thrombus, it is unlikely to recognize or correctly analyse it. In contrast, a clinician drawing on prior knowledge and experience would still be able to interpret such findings.

Where possible, EF should be measured quantitatively using Simpson’s biplane method; however, some studies relied on visual estimations.^51^ In studies that presented Bland–Altman plots comparing visual and biplane methods, the biplane measurements demonstrated tighter clustering patterns. Although similar clustering in other studies implies the use of the same method, this cannot be conclusively determined.

## Conclusion

AI-derived GLS, and to a lesser extent 3D EF, demonstrate reproducibility equal to or exceeding clinicians, positioning GLS as the most reliable entry point for clinical AI adoption. Cautious interpretation of 2D EF is warranted due to its greater variability, which may affect reproducibility and lead to inconsistencies in clinical decision-making.

## Supplementary Material

ztaf145_Supplementary_Data

## Data Availability

All data analysed in this study are derived from previously published sources, which are cited within the article and its supplementary materials. Additional details are available from the corresponding author upon reasonable request.

## Appendix 1 Medline via ovid search strategy

1. exp Echocardiography/

2. echocardiog*.tw.

3. (Cardiac adj3 echo).tw.

4. 1 or 2 or 3

5. exp Artificial Intelligence/

6. (‘algorit*’ or ‘artificial intelligence’ or ‘computational intelligence’ or ‘machine intelligence’ or ‘computer reasoning’ or ‘computer vision*’ or ‘machine learnin*’ or ‘deep learnin*’ or ‘neural network*’ or ‘computer-assisted’ or ‘computer-aided’ or ‘support vector machine*’ or ‘support vector network*’ or ‘computer methodologies’ or ‘automated pattern recognition’ or ‘pattern recognition systems’ or (comput* and ‘pattern recognition’) or ‘k nearest neighbour’ or kNN or ‘random forest’).tw.

7. 5 or 6

8. (reproducib* or reliab* or repeatability or replicability or precision or consistency or variability).tw.

9. 4 and 7 and 8

## Appendix 2 Formulae for inverse-variance weighted meta-analysis of bias

1. Bias, lower LoA, upper LoA, and sample size (*n*) for each individual study were imported into SPSS.

2. Calculate standard deviation of differences (SD*d*) for each study:

$$\mathrm{SD}d=\frac{\mathrm{Upper}\;\mathrm{LoA}-\mathrm{Lower}\;\mathrm{LoA}}{3.92}$$

3. Log-transform variance:

$$\mathrm{Log}\;\mathrm{of}\;\mathrm{Variance}=\frac{2}{n-1}$$

4. Compute weights of inverse variance:

$$\mathrm{Weight}=\frac{1}{\mathrm{Log}\;\mathrm{of}\;\mathrm{variance}}$$

5. Compute weighted bias:

WeightedBias=WeightxBias

6. Sum weights and weighted bias:

SumofWeights=∑Weights

SumofWeightedBias=∑Weightedbias

7. Calculate the inverse-variance weighted bias:

$$\mathrm{Pooled}\;\mathrm{mean}\;\mathrm{bias}=\frac{\mathrm{Sum}\;\mathrm{of}\;\mathrm{Weighted}\;\mathrm{Bias}}{\mathrm{Sum}\;\mathrm{of}\;\mathrm{Weights}}$$

8. Compute SE of the mean bias:

$$\mathrm{SE}\;\mathrm{of}\;\mathrm{Bias}=\frac{\mathrm{SDd}}{\sqrt{n}}$$

9. Compute SE of the inverse-variance weighted bias:

$$\mathrm{SE}\;\mathrm{of}\;\mathrm{Pooled}\;\mathrm{Mean}\;\mathrm{Bias}=\;\sqrt{\frac{1}{\mathrm{Sum}\;\mathrm{of}\;\mathrm{Weights}}}$$

10. Compute 95% CI for the inverse-variance weighted bias:

LowerCI=Bias−(1.96xSEofBias)

UpperCI=Bias+(1.96xSEofBias)

## Appendix 3 Heterogeneity estimates from random-effect meta-analyses

**Table:**

Random-effect meta-analysis	I² (%)
Bland–Altman 2D EF^a^	99.00
Bland–Altman 3D EF^a^	98.00
Bland–Altman 2D GLS^a^	98.00
Inter-observer ICC 2D EF	93.00
Intra-observer ICC 2D EF	100.00
Inter-technique ICC 2D EF	97.00
Inter-observer ICC 2D GLS	45.00
Intra-observer ICC 2D GLS	87.00
Inter-technique ICC 2D GLS	98.00

^a^Bland–Altman *I*² values are derived from the meta-analyses of the upper limits of agreement (LOA). Similar values would be expected for the lower LOA and bias.

## Appendix 4 Summary of CLAIM domain scores across included studies

**Table:**

Section	Mean percentage (%)
Title/abstract	100.00
Introduction	100.00
Study design	100.00
Data	69.17
Ground truth	94.74
Data partitions	64.91
Model	73.68
Training	45.61
Evaluation	57.89
Data	60.53
Model performance	78.95
Discussion	100.00
Other information	35.09
