# Supplementary material for: Reproducibility of echocardiographic measurements of left ventricular systolic function: a systematic review and meta-analysis comparing artificial intelligence and clinician estimates
Source: Eur Heart J Digit Health. 2025 Dec 11;7(3):ztaf145. doi: 10.1093/ehjdh/ztaf145 (PMC12994475; doi:10.1093/ehjdh/ztaf145)
Supplement: ztaf145_Supplementary_Data [file ztaf145_supplementary_data.docx]

**Supplementary material**

**Supplementary Table 1**

**Supplementary Table 1**: Raw mean Ejection Fraction (EF) values with standard deviation (SD) as reported by Clinicians and Artificial Intelligence (AI) across included studies.

| **2D/3D** | **Study** | **Clinicians’ mean EF ± SD (%)** | **AI mean EF ± SD (%)** |
| --- | --- | --- | --- |
| 2D | Asch et al. (2019) (37) | Not reported. | Not reported. |
| 2D | Mor‑Avi et al. (2023) (30) | 60.4 ± 2.6 | 61.9 ± 6.3 |
| 2D | Morbach et al. (2024) (29) | 59.8 ± 4.8 | 59.9 ± 4.9 |
| 2D | Olaisen et al. (2024) (25) | Dataset 1 (real-time, 50 patients) = 52.8 ± 10.7  Dataset 2 (HUNT4 reproducibility, 40 patients) = 57.1± 6.2  Dataset 3 (HUNT4, 1881 patients) = 59.1 ± 6.6  Dataset 4 (Tromsø7, 849 patients) = 55.1 ± 7.1 | Dataset 1 (real-time, 50 patients) = 50.9 ± 9.7  Dataset 2 (HUNT4 reproducibility, 40 patients) = 54.2 ± 7.3  Dataset 3 (HUNT4, 1881 patients) = 53.6 ± 6.8  Dataset 4 (Tromsø7, 849 patients) = 55.4 ± 6.4 |
| 2D | Lin et al. (2024) (40) | Not reported. | Not reported. |
| 2D | Jang et al. (2024) (36) | 51.81 ± 9.31 | 51.37 ± 9.81 |
| 2D | Knackstedt et al. (2015) (33) | Local centre: 57 ± 10.8  Reference centre: 56.5 ± 12.3 | 56.8 ± 12.5 |
| 2D | Lafitte et al. (2025) (41) | 55.7 ± 10.7 | 55.8 ± 12.1 |
| 2D | Jiang et al. (2023) (35) | 60.7 ± 4.9 | 61.6 ± 5.7 |
| 2D | Li et al. (2025) (39) | Coronary heart disease: 56.80 ± 9.87  LV hypertrophy: 59.60 ± 10.54  Cardiac amyloidosis: 53.36 ± 10.71  Preserved EF: 58.23 ± 10.09  Pericardial effusion: 57.23 ± 10.48  Mitral regurgitation: 55.37 ± 10.11  Arrhythmia: 57.81 ± 10.00 | 57.0 ± 8.0 |
| 2D | Kim et al. (2022) (34) | 67.0 ± 4.9 | Not reported. |
| 2D | Sveric et al. (2023) (38) | Not reported. | Not reported. |
| 2D | Myhre et al. (2024) (27) | 57.63 ± 6.65 | 63.31 ± 7.73 |
| 2D | Myhr et al. (2018) (28) | 54 ± 12 | 61.6 ± 11.9 |
| 3D | Myhre et al. (2024) (27) | 59.40 ± 6.11 | 59.40 ± 6.11 |
| 3D | Myhr et al. (2018) (28) | 60.2 ± 13.1 | 60.2 ± 13.1 |
| 3D | Medvedofsky et al. (2018) (31) | 39 ± 15 | 40 ± 15 |

**Supplementary Table 2**

**Supplementary Table 2:** Raw mean Global Longitudinal Strain (GLS) values with standard deviation (SD) as reported by Clinicians and Artificial Intelligence (AI) across included studies.

| **Study** | **Clinicians’ mean GLS ± SD (%)** | **AI mean GLS ± SD (%)** |
| --- | --- | --- |
| Nyberg et al. (2024) (26) | Recording 1:  Observer 1: 17.2 ± 3.0  Observer 2: 17.5 ± 2.6  Observer 3: 18.3 ± 3.2  Recording 2:  Observer 1: 17.2 ± 3.0  Observer 2: 17.1 ± 2.9  Observer 3: 18.4 ± 3.2 | Recording 1: 15.9 ± 2.5  Recording 2: 16.0 ± 2.5 |
| Rogstadkjernet et al. (2024) (23) | Not reported. | Not reported. |
| Salte et al. (2023) (22) | Dataset I: ranged from −17.2 ± 3.0 to −20.1 ± 3.2 (across the four readers).  Dataset II: ranged from −17.7 ± 2.6 to −19.2 ± 2.7 (across the four readers). | Dataset I: −16.0 ± 2.4  Dataset II: −16.8 ± 2.7 |
| Kuwahara et al. (2024) (32) | −15.9 ± 3.4 | −19.0 ± 3.7 |
| Jang et al. (2024) (36) | −13.3 ± 4.0 | −11.9 ± 3.3 |
| Knackstedt et al. (2015) (33) | − 21 ± 6.0 | −19.1 ± 6.0 |
| Lafitte et al. (2025) (41) | A2C: –16.8 ± 3.9  A3C: –17.0 ± 2.9  A4C: –16.9 ± 4.2 | A2C: –17.4 ± 5.0  A3C: –17.6 ± 5.3  A4C: not reported. |
| Jiang et al. (2023) (35) | −19.1 ± 2.0 | −17.9 ± 2.2 |
| Myhre et al. (2024) (27) | −18.3 ± 3.4 | −22.1 ± 3.6 |
